# Assessment of Comfort Variation among Different Types of Driving Agricultural Tractors: Traditional, Satellite-Assisted and Semi-Automatic

**DOI:** 10.3390/ijerph17238836

**Published:** 2020-11-27

**Authors:** Elio Romano, Carlo Bisaglia, Aldo Calcante, Roberto Oberti, Alberto Zani, Denis Vinnikov, Andrea Marconi, Ermanno Vitale, Massimo Bracci, Venerando Rapisarda

**Affiliations:** 1 Council for Agricultural Research and Economics, Research Centre for Engineering and Agro-Food Processing, 24047 Treviglio, Italy; elio.romano@crea.gov.it (E.R.); carlo.bisaglia@crea.gov.it (C.B.); 2 Department of Agricultural and Environmental Sciences-Production, Landscape, Agroenergy, Università di Milano, 20133 Milan, Italy; aldo.calcante@unimi.it (A.C.); roberto.oberti@unimi.it (R.O.); alberto.zani1@unimi.it (A.Z.); 3 Department of Epidemiology, Biostatistics and Evidence-based Medicine, al-Farabi Kazakh National University, Almaty 050040, Kazakhstan; denisvinnikov@mail.ru; 4 Occupational Medicine, Department of Clinical and Experimental Medicine, University of Catania, 95123 Catania, Italy; dott.marconi@yahoo.it (A.M.); vrapisarda@unict.it (V.R.); 5 Occupational Medicine, Department of Clinical and Experimental Medicine, Polytechnic University of Marche, 60126 Ancona, Italy; m.bracci@email.it

**Keywords:** comfort evaluation, electromyography, pressure sensors

## Abstract

Over the past years, in the agricultural field, geo-localization has been introduced in order to develop specific farming processes, optimize resources, and reduce environmental pollution. Researchers have found alternative driving methods to traditional ones, such as assisted and semi-automatic driving. The aim of this study was to monitor the musculoskeletal efforts necessary to carry out different kinds of driving. The muscular strain was assessed using surface electromyographic devices, the distribution of the pressure exerted by the operator’s body on the seat was observed by using two barometric pads applied on the seat back and on the seat, respectively, while the body movements and postures were analyzed through a Microsoft Kinect Camera 3D acquisition system. Results showed a significantly greater muscular activation during manual and assisted driving conditions. The pressure exerted by the operator on the barometric pads was significantly higher in manual and semi-automatic driving modes than in the assisted one. A remarkable increase in the average swinging speed of examined joints was also detected, as well as the distances run by the joints in semi-automatic driving. From our study, assisted driving seems to be the best driving mode both in terms of joint economy and from the efficiency of agricultural processes.

## 1. Introduction

In recent years, the accomplishment of agricultural operations carried-out with tractors has been heading towards the integration of traditional mechanics with geolocation technologies, to bring farming procedures closer to the precision agriculture logic [1,2]. The possibility to control the tractor steering by using Global Navigation Satellite Systems (GNSS) is an important contribution to improving the efficiency of agricultural practices [3,4] and allows savings of time, fuel, labor, and production factors contributing to the economic and environmental sustainability of the agricultural process [5,6]. However, today, driving agricultural vehicles still requires great concentration and repeated gestures to obtain quality and maintain security conditions [7]. In fact, the operator, depending on the type of farming operations, needs to move levers, turn the wheel, press pedals, turn their back to check the machine and the work done [8,9]. Therefore, tractor drivers are exposed to noise, vibrations, and possible incorrect postures that can generate musculoskeletal disorders [10,11].

Investigations conducted on farms have shown that the tension on the forearms and neck during some hours of work as a hauler may be associated with discomfort and pain in the upper limbs and neck [12].

Agricultural machinery operators report performance alteration usually associated with back pain and sitting disorders [13,14,15]. Mayton et al. (2008) [16] found that 24% of tractor drivers performing different agricultural operations reported neck symptoms, and 96% of them reported bending or twisting their necks at work; 64% of tractor operators reported back symptoms, including pain and stiffness.

The new technologies, based on GNSS and mechatronic components, aim to help the driver to conduct tractors and self-propelled machines along pre-defined paths. This, from the ergonomic point of view, allows the operations to be simplified, reducing the number of incorrect or uncomfortable postures and improving the awareness of the operator. The most widespread GNSS-controlled driving devices are the assisted and the semi-automatic driving systems.

Assisted driving enables the operator to follow parallel and equidistant tracks shown on a display positioned on dashboard of the tractor. An appropriate software virtually processes the geo-localized information provided by the GNSS receiver positioned on the top of the tractor’s cabin and draws guidelines on the screen representing the field to avoid unnecessary overlap and optimize work paths.

In the case of semi-automatic driving, the operator only has to turn the steering during the turning maneuvers at the end of the field, whereas the automatic control systems that act on the steering wheel or on the front axis set the path along the pre-defined line during the rest of the path [17].

The aim of this study was to evaluate the operator’s comfort with electromyography and to study the operator’s driving posture using three different types of driving system: manual, satellite-assisted, and semi-automatic.

## 2. Materials and Methods

In order to study the operator’s comfort, we measured: (1) barometric body pressures onto the seat; (2) muscular activation by electromyography; and (3) posture analysis by a set of motion sensing devices.

During this study, a series of experimental tests using three different guidance systems (manual, satellite-assisted, and semi-automatic) were conducted to take into account the same agricultural operation (harrowing).

Tests were performed in July 2018 on a 3-hectare land strip previously cultivated for the production of wheat (45°31′14″ N, 9°34′18″ E), located in Treviglio (BG, Italy) on a farm near CREA headquarters (Council for Agricultural Research and Economics). The soil was homogeneous for physical characteristics [8]: sand = 68%; silt = 24%; clay = 8%. Stone represent 1/3 of the test field. During the tests, the median soil humidity was 18%, and the median resistance to penetration was 0.98 MPa.

All procedures performed in present study were in accordance with the 1964 Helsinki Declaration and its later amendments or comparable ethical standards. All participants gave their signed consent to participate in the study. The study was approved by the ethics committee of University of Catania number code 321/18.

Experimental tests included a crop-harrowing operation, carried out with a spring harrow (MAINARDI 400, Mainardi Machine Agricole, Cremona, Italy) coupled to a 120-kW (JOHN DEERE 6920s, John Deere, Moline, IL, USA) variable transmission tractor, equipped with an S-Guide RTK (StarFire6000, John Deere, Moline, IL, USA) automated steering system. Harrowing was observed on a path, which included four straights lines of 150 m connected by three turns. This route was carried out in three driving conditions: (a) manual, without support of the satellite system, (b) with satellite activated only for assistance on the display through guidelines and manual correction of the path and (c) semi-automatic, which required operator intervention on the steering only for the turn phase and never during the straight line.

**Figure 1:**
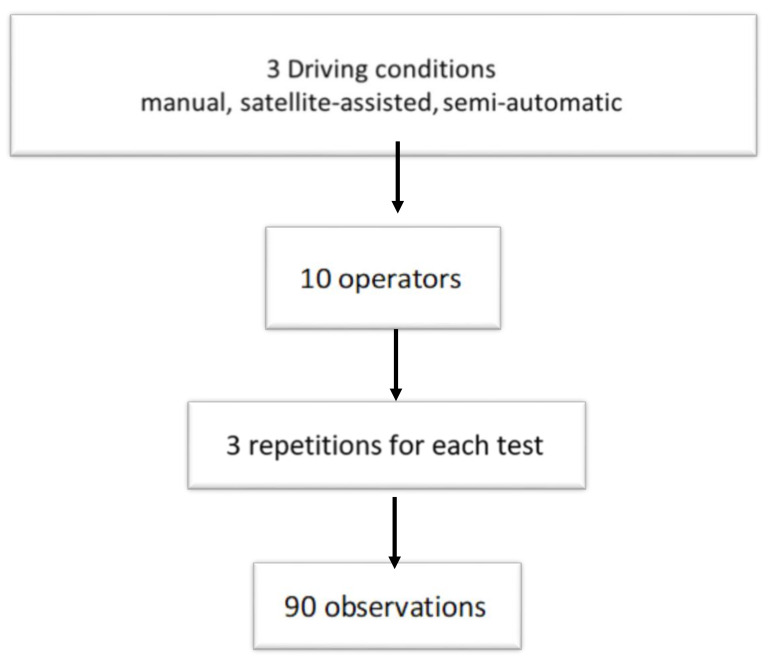
Experimental scheme of the study.

The tests were conducted by 10 operators, male, right-handed, average age 33.1 ± 5.7 years, healthy volunteers, with the same body-size (height: 1.80 ± 0.02 m, weight: 80 ± 1 kg, BMI: 24 ± 0.7 kg/m^2^), with five-year experience in the agricultural practices of soil processing. Figure 1 shows three driving conditions (manual/satellite-assisted/semi-automatic) × 10 operators × 3 repetitions (successive in a single processing), with 90 observations altogether.

With a time interval of one hour, every operator carried out one test without the support of the satellite system (manual), one test with driving assisted by the satellite system (assisted), and the last test using the automatic guidance system (semi-automated mode).

**Figure 2:**
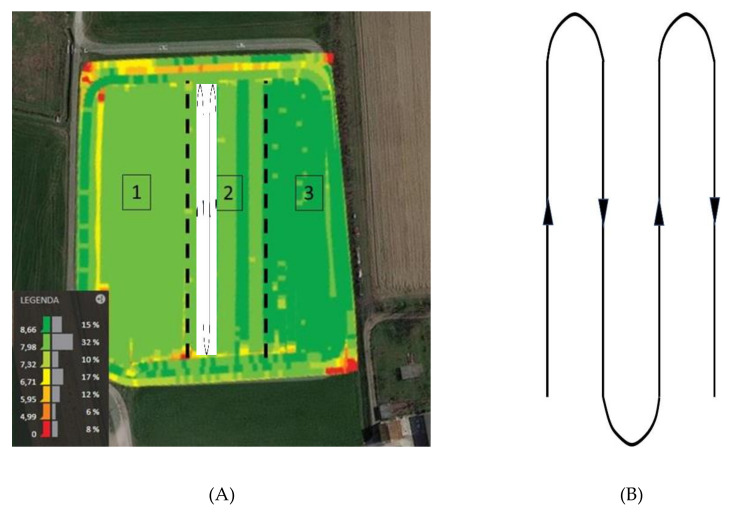
Test surface with a false-color map representing harrowing speeds in the three driving modes: (1) manual, (2) satellite-assisted, and (3) semi-automatic. Path of the measured parameters. (**A**) Test field (**B**) Path in which each acquisition was developed.

During all of the harrowing phases, the satellite system provided storable geolocation data (Figure 2).

### 2.1. Satellite System

The StarFire 6000 double frequencies receiver with the RTK (Real-Time Kinematic) correction is capable of calculating the tractor position—with centimetric accuracy—the speed, and the current time, to ensure proper operation of the considered guidance systems. The same data were transmitted and collected by the farm information system for further elaboration.

### 2.2. Muscular Engagement Detection System—Electromyograph

We tested muscular engagement with surface electromyography (EMG) and measured the mean frequency (MNF) of the signal, the most useful and popular frequency-domain features [18] and frequently used for the assessment of muscle fatigue in surface EMG signals [19,20]. MNF is an average frequency that is calculated as the product sum of the EMG power spectrum and the frequency divided by the total sum of the power spectrum [20]. MNF has a similar definition of several features, i.e., the central frequency (cf), centroid, and the spectral center of gravity, shown in several studies [21,22]. In addition, MNF is also called mean power frequency and mean spectral frequency in several works. The definition of MNF is given by (1):

(1) $$\mathrm{MNF}\;=\frac{\sum_{j=1}^{M}f_{j}P_{j}}{\sum_{j=1}^{M}P_{j}}$$

where *f_j_* is the frequency value of EMG power spectrum at the frequency bin *j*, *P_j_* is the EMG power spectrum at the frequency bin *j*, and M is the length of the frequency bin. In the analysis of the EMG signal, M is usually defined as the next power of 2 from the length of EMG data in the time-domain.

**Figure 3:**
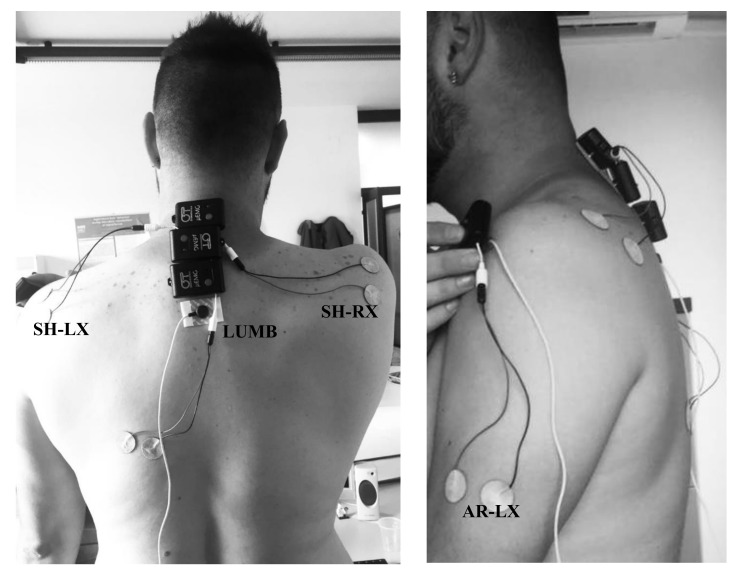
The position of the electrodes for electromyography.

A system was used to measure muscle electro-signals in real-time during the trial, and the acquisition was elaborated to calculate the MNF by the muscles of the left forearm (AR-LX), the right (SH-RX) and left (SH-LX) shoulder, and the vertebral column (LUMB) (Figure 3).

Four portable and lightweight EMG data loggers with an internal lithium ion battery (OT Bioelectronics-OTB, Turin, Italy) were used with adhesive electrodes with a diameter (ø) = 24 mm, in which the sensor surface (in Ag/AgCl) was 80 mm^2^, with an adhesive area of 1808 mm^2^ with a thickness of 1 mm. We applied sensors on a subject prior to the test. The electromyographic sensors are disposable electrodes that are fixed on the cleansed skin in the projection of involved muscles, including left arm, right and left shoulder, and back lumbar area.

For each muscle, two sensors were fixed at about 0.01 m next to each other and according to the direction of the maximum length of the fibers. Finally, when the mass was placed, an electrode was attached at the level of the bone as tightly as possible, whose potential value was taken as reference for the values of tension acquired by other sensors. The reference electrode was fixed under the nape, corresponding to the first thoracic vertebra (T1). Muscle fatigue is reflected in a significant increase of the EMG signal amplitude and in a shift of the power spectrum towards low frequencies [23,24]. The increase in amplitude can be attributed to neuromuscular recruitment of additional motor units and/or increased spatial and temporal synchronization of such units.

**Figure 4:**
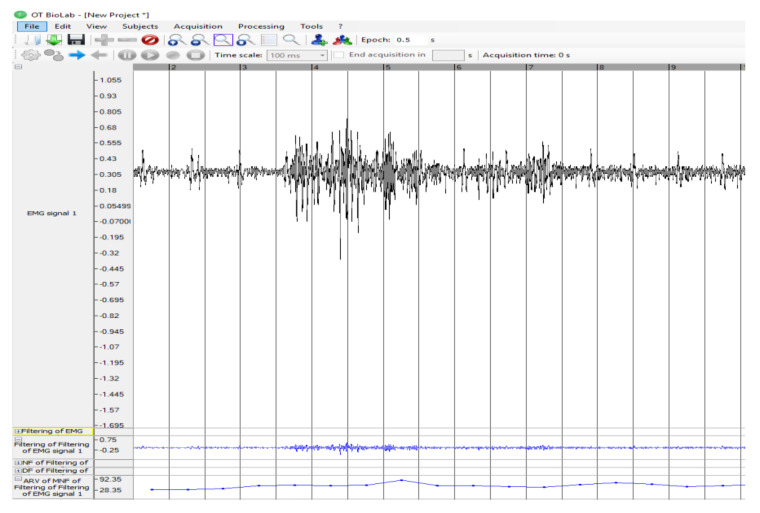
The interface of the software used to process sEMG signal with an example of the EMG signal 1 (black line), and derivation of the ARV (second blue line from the bottom), and the filtered signal (blue bottom line). On the abscissa the time in seconds.

A greater concentration of the spectrum around the low frequencies can instead be associated with the increase in time, in which each single motor unit (motor neuron and the skeletal muscle fibers innervated by that motor neuron’s axonal terminals) remain active and with the consequent reduction in conduction speed. We applied data elaboration with OT BioLab 1.7 Bioelectronics software (Figure 4), a band-pass filtering that cut the frequencies outside the range 10–450 Hz, because below 10, they constitute background noise and above 450 they are not guaranteed reliable by the instrumentation used. The filtered signal was, therefore, suitable for the subsequent phase of signal rectification (full-wave rectification) when all the negative amplitudes were converted into positive, i.e., the absolute signal value was calculated. These steps were necessary to obtain amplitude parameters from the curve, such as the mean, peak value, and root mean squared (RMS). The amplitude of the EMG signal is influenced by the number of active motoneurons and by the form and speed of the activation potential. The signal amplitude can be estimated as the average value of the rectified signal (ARV, average rectified value). Normalization, with respect to the maximum voluntary contraction (MCV), was detected by each operator before the tests by performing a complete extension of the observed muscle, free, without constraints or loads, which made it possible to calculate the number of muscular involvement occurrences.

### 2.3. Barometric Detection System

**Figure 5:**
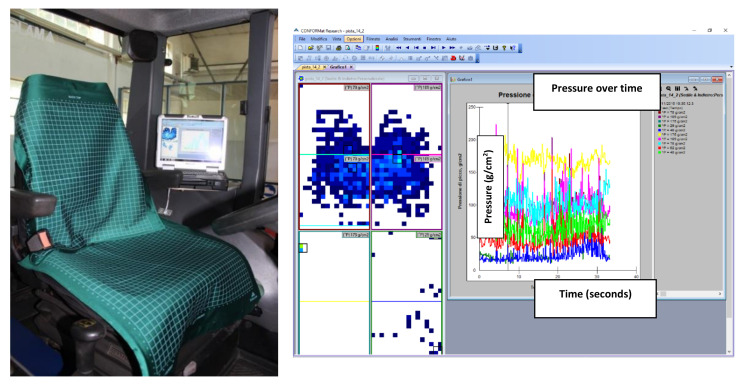
Sensor mat for barometric measurements (CONFORMat Seat) (**left**); a picture of the acquired signal after one test (**right**).

The barometry was observed through two sensorized mats (Figure 5) consisting of a grid of 32 × 32 load cells (1024 sensors/carpet), able to provide the distribution map of decomposed dynamic load per cell in real-time, applied as one to the back and one to the seat. The sensor consisted of a carpet of resistive sensors (32 × 32) [25]. The instrumental chain consisted of two Evolution Handle data scanners (Tekscan Pressure Measurement System, 1998–2012, South Boston, MA, USA) with a 100 Hz scan rate. The sensor was 0.64 mm thick and measured a pressure range between 0 and 1000 g/cm^2^ (98 kPa). Each pressure profile was called a frame. From each frame, the maximum and mean values could be calculated.

As previously described [7], CONFORMat Research ver. 7.60-21C software (Tekscan Pressure Measurement System, 1998–2012, South Boston, MA, USA) was used to read the data collected with the sensor array. This was a capacitance sensor system, which received the maximum effective pressure distribution relief while the subjects were seated.

The software was also able to provide graphs illustrating the pressure, the contact area, and the distribution of force overtime and could execute the dynamic playback of two or more signals simultaneously. In addition, the system could export data in ASCII code.

From each single test, the following values were derived from carpet sensors: max pressure peak (P_max_); average pressure value (P_avg_); average percentage of cells activated by pressure values ranging between 130–400 g/cm^2^ (12.7–39.2 kPa) (NC_130–400_) as indicated in a previous study [26]. P_max_ is the mean value of maximum pressure peaks recorded by each frame in each single test. P^agv^ is the average value of all pressure peaks recorded by each frame for each single test. NC_50–130_, NC_131–400,_ and NC_401–1000_ values were calculated with the Equation (2):

(2) $$\%NC^{\left(range\right)}=\frac{N_{S\left(range\right)}}{N_{S}}*100$$

where:

*N_S_*_(*range*)_: number of sensors measuring a pressure between 130–400 g/cm^2^ (12.7–39.2 kPa),
*N_S_*: number of active sensors by a pressure > 0 g/cm^2^ (>0 kPa).

### 2.4. Posture Detection System–Kinect

The posture was studied through a three-dimensional Microsoft Kinect camera that is a line of motion sensing device produced by Microsoft as an accessory for interactive control of video game console.

Kinect is a composite measuring device that delivers RGB-D output, i.e., a color image and a correspondent depth image of the scene. Because of its low cost, reliability, and availability of development software libraries, Kinect has become a state-of-the-art 3D sensor widely employed in research applications.

In this study, the Kinect V2 version was used. Kinect V2 captures RGB images with a resolution of 1920 × 1080 pixels and depth-images with a 512 × 424 pixels resolution. The depth-images are acquired by a time-of-flight emitter-receiver module, able to measure the distance from the sensor by evaluating the time needed by an IR pulsed illumination source to travel from emitter to target.

As a result, a 512 × 424 array of points to sensor distance, i.e., the depth image, for a field of view of 70° horizontally and 60° vertically was obtained. Kinect V2 has an operative distance range of distance measurements between 0.5 m and 7 m with a typical accuracy in the range of 3 mm for distances below 1.5 m [27].

**Figure 6:**
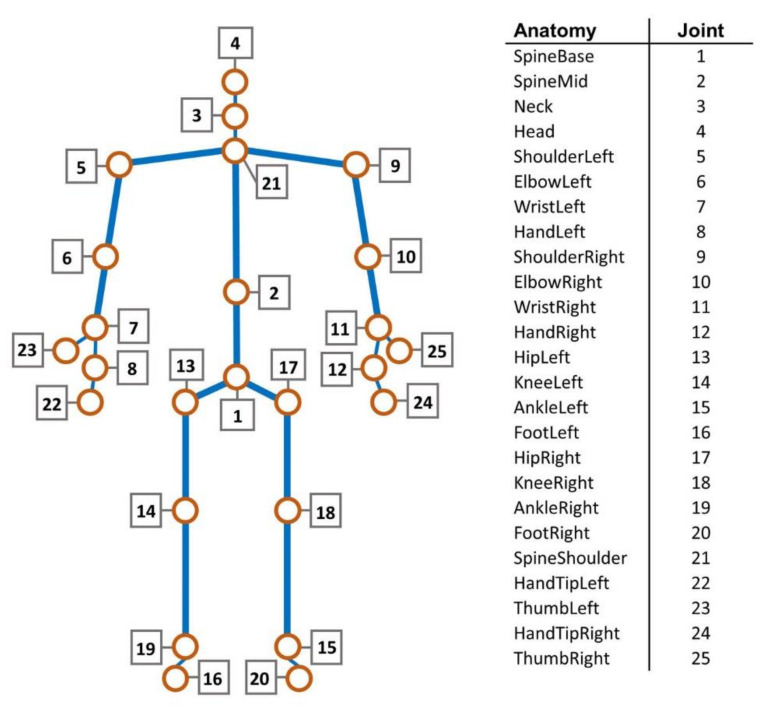
The Kinect 3D skeleton and the anatomical parts corresponding to every joint point.

By using Kinect intrinsic parameters and perspective projection relationship, a depth image can be mapped into a point cloud (x, y, z) and referred to as a coordinate system originating from the camera position. Furthermore, through a calibrated linear transformation, color data from the corresponding RGB image can be included to obtain a colorized point cloud (x, y, z, r, g, and b) [28]. Being designed for user-console interaction through gestures and spoken commands, Kinect V2 software libraries enabled us to extract and track the positions of the human figure by capturing the 25 most representative joint points of the human body (Figure 6) starting from its depth map. Each joint is identified by a 3D position (x, y, z). X is the horizontal axis, y is the vertical axis, and z is the distance between the joint and the Kinect sensor. This allowed us to create the 3D skeleton monitoring systems data in order to detect and evaluate the activity of the framed human body [29,30].

**Figure 7:**
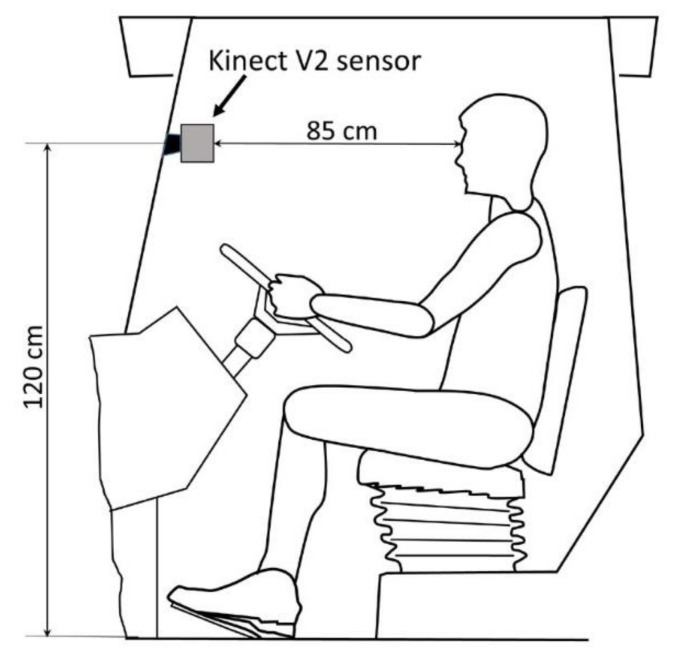
Scheme of the tractor cabin with the Kinect sensor position.

In the present study, a Kinect V2 sensor was mounted in the cabin of a John Deere 6920s tractor in front of the tractor driver, as shown in Figure 7. The sensor was fixed to the cab screen glass through a suction cup holder, 1.20 m high from the cabin floor, 0.85 m away from the driver’s eyes. This position was chosen in order to record the driver’s position during driving operations, with the aim to detect any possible differences in their posture attitude when using the different driving systems considered.

In order to acquire tractor driver’s images during the field tests, a dedicated acquisition software was developed in Matlab 2019a (MathWorks Inc., Natick, MA, USA) and implemented on a rugged notebook placed in the tractor cabin. The software allowed to continuously record RGB and depth images of the tractor driver with a frequency of 3 Hz and process the depth images to obtain the 3D skeleton in real-time.

Joint points considered to evaluate the posture of the driver were:

(a) neck: joint 3;
(b) head: joint 4;
(c) left shoulder: joint 5;
(d) right shoulder: joint 9.

The posture of the tractor’s driver was analyzed in two different ways:

(1) in terms of average shifts and standard deviation computed starting from the data acquired during the field tests [31,32].

In particular, being *j*-th the number of the joint observed, for the *j*-th joint, relative shifts *d_j_(t)* at a given time *t* was calculated as the distance covered by the *j*-th joint (3):

(3) $$d_{j}\left(t\right)=\;\sqrt{(x_{j}{\left(t+\Delta t)-x_{j}\left(t\right)\right)}^{2}+(y_{j}{\left(t+\Delta t)-y_{j}\left(t\right)\right)}^{2}+(z_{j}{\left(t+\Delta t)-z_{j}\left(t\right)\right)}^{2}}$$

where x, y, z are the three coordinates of each considered joint points: 3, 4, 5, and 9.

The joint speed *v_j_(t)*, expressed in cm/s, was calculated by dividing *d_j_(t*) by the time interval between two acquisitions and the obtained values were averaged for each considered driving condition.

(2) The 3D coordinates (x, y, and z) of the joints 3, 4, 5, and 9 were compared to the coordinates of the same joints measured with the driver in a resting position (i.e., with the upright head, the bust correctly rested against the seat back and with the hands on the steering wheel). The accumulated distance (AcD) observed between the driver’s resting position and the positions kept during the field tests was expressed, for the four considered joint points, as (4):
  (4) $$d_{j}=\overset{j=1}{\underset{n}{\sum }}\sqrt{{\left(x_{j}-x_{rp}\right)}^{2}+{\left(y_{j}-y_{rp}\right)}^{2}+{\left(z_{j}-z_{rt}\right)}^{2}}$$
  where *x_j_*, *y_j_*, *z_j_* are the three coordinates of each considered joint points, *x_rt_*, *y_rt_*, *z_rt_* are the three coordinates of the same joint point measured at the tractor driver’s resting position, and N is the number of observations measured during the field tests for each driving mode. Also, in this case, the obtained values were averaged for every system.

Since the turning maneuvers at the end of a field must be carried out manually by the driver regardless of the driving system used, only the parallel and straight paths were considered, disregarding the movements of the driver during the turnings.

### 2.5. Statistical Analysis

Data analyses were conducted with the Comprehensive R Archive Network (CRAN) software (Institute for Statistics and Mathematics, Wien-Umgebung, Austria). Data were reported as average (M) ± standard deviations (SD).

Data from the electromyographic, barometric, and postural acquisition systems, as well as those related to geolocation, were processed through descriptive, statistical, and inferential analysis. We tested the normality of distribution and variances homogeneity.

We applied the analysis of variance (ANOVA) in order to see whether between-group variability exceeded within-group one.

In this study, this analysis was used to find significant differences in three groups that represented three driving modes: traditional, assisted, and semi-automatic. The analysis aimed to highlight the significance of the effect of the driving system with respect to the other variability factors, i.e., the change of operator and the repetition along the path. The analysis of variance was applied to all response values obtained in the tests, such as the number of electromyograms close to maximum voluntary contraction, the mean pressure, pressure peak, and the number of stimulated load cells in a range between 130 and 400 g cm^−2^ (12.7–39.2 kPa), regarding barometric test results, values obtained from the reading with Kinect, for the postural study and the average values of harrowing speed. Furthermore, we also tested correlations between all obtained values in order to detect any intercorrelation. Whenever data were normally distributed and variances were homogenous, we used parametric methods.

Regarding the driving system, Tukey’s test for multiple comparisons was applied. For all tests, a significance level of *p* ≤ 0.05 was chosen.

## 3. Results

The satellite system collected space-time information, which showed an average harrowing speed of 8.20 ± 0.67 km/h for the entire three-hectare test area. However, the harrowing was carried out at different speeds in three driving modes. Thus, recorded speed was 8.03 ± 0.23 km/h in manual mode; 7.93 ± 0.16 km/h in assisted driving mode; and 8.66 ± 0.79 km/h in semi-automatic mode, significantly greater compared to other modes (*p* < 0.05). Table 1 and Table 2 indicate electromyographic and barometric systems outcomes.

ANOVA showed statistically significant (*p* < 0.01) differences in values of electromyographic and barometric tests with different guide systems. In particular, on the left arm (AR_SX), the muscular strain was greater in manual driving than in assisted driving and statistically significantly in semi-automatic modes, just as muscular strain was significantly greater in assisted driving compared to semi-automatic modes. Muscular strain assessed via muscle activation in two shoulders (SH_SX and SH_DX) was significantly greater in manual driving than in semi-automatic driving modes (Table 1). Finally, muscular strain assessed with the electromyograph placed on the spine (LUMB) was greater in the manual driving activity than in the assisted and semi-automatic ones, just as muscular strain was greater in assisted driving compared to semi-automatic (Table 1).

Regarding pressure exerted by the operator on the seat, Pavg was significantly higher in manual driving than in assisted and semi-automatic ones. In addition, Pmax was significantly higher in the manual driving than in the assisted one. Similarly, pressure cells activation (NC130_400) was significantly greater in the manual driving compared to the assisted one. No statistically significant influence was detected in MNF and barometric pressure values in the repetitions and between operators.

Table 3 summarizes the average speed of every considered joint in three different guidance methods measured by using the Kinect V2 sensor.

The differences between average speeds (Table 4) measured in joints 3 (neck) and 4 (head), respectively, were similar in traditional and assisted driving; but significantly lower than in automatic driving. However, the average speed in joints 5 (left shoulder) in traditional driving was significantly less compared to automatic driving. Finally, no significant difference was observed between the three driving modes with regard to the average speed in joints 9 (right shoulder).

The average AcDs measured in joints 3 (neck) and 4 (head) were similar in traditional and assisted driving, but significantly lower than in automatic driving. However, the average AcD measured in joint 5 (left shoulder) was significantly lower in traditional driving than the automatic one. Finally, no significant difference in the average AcD measured in joint 9 (right shoulder) was observed in three driving modes.

**Figure 8:**
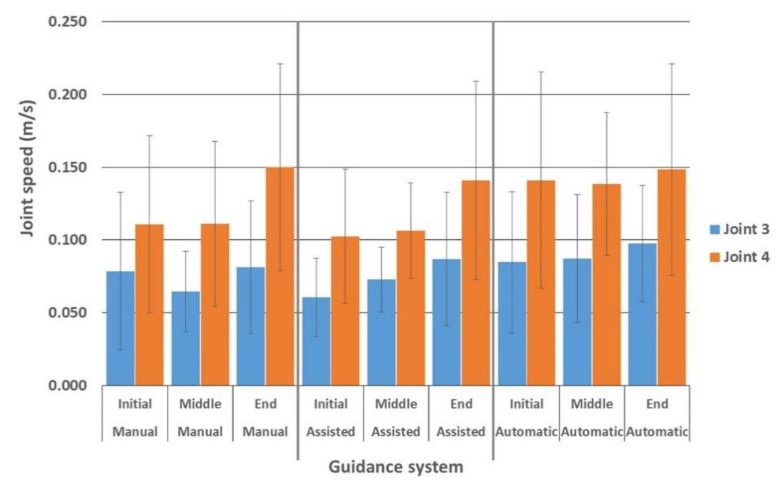
Speed values measured at the beginning, during, and at the end of the field tests carried out using the three analyzed guidance systems. Letters (a, b and A, B) refer to Tukey’s test on the two joints.

Furthermore, when driving manually, neck speed was reduced in the median phase, but increased again towards the end. In assisted driving, neck speed increased during the shift with a significant increase at the end of the test. When using the automatic driving system, the neck’s joint speed was almost constant during the entire field test (Figure 8).

Regarding joint 4 activity, in manual and assisted driving, head speed progressively increased from the initial phase through the end with a significant difference between the starting/middle phases and the final moments in both driving modes (Figure 8). On the contrary, the head’s joint speed was almost constant during the entire field test in the automatic driving mode.

## 4. Discussion

The introduction of new tractor driving methods in farming has posed new issues about risks for safety and health in agricultural drivers [33]. As a matter of fact, a previous study [8] had already observed that driving activity, considering several tractor-uses and mounting, could entail a greater musculoskeletal strain with an early lack of comfort at first, but with the occurrence of real pathologies in the most stressed osteoarticular areas in the long run [15].

We found that the tractor moved at a significantly higher speed during semi-automatic driving compared to the traditional (manual) and semi-automatic modes.

The muscle strain of the upper limbs (MNF values) and the pressure exerted by the operator on the seat (barometric values) were significantly higher in the traditional (manual) driving mode as opposed to the alternative modes (assisted and semi-automatic). In addition, differences in MNF and barometric values were also detected comparing two new driving systems (assisted vs. semi-automatic).

Manual driving always involves a significantly greater muscle commitment of the left upper limb (AR_SX), of the cingulum-shoulder-humeral, and of the paravertebral muscles of the column of the cervicothoracic tract compared to semi-automatic driving. In assisted driving, left arm (AR_SX) muscular strain was lower than in manual driving but was significantly greater than in semi-automatic, just as muscular strain was significantly greater in manual driving compared to semi-automatic. Regarding muscle activation of the two shoulders (SH_SX and SH_DX), the muscular effort was significantly greater in manual than in semi-automatic driving. A similar pattern was observed for the spine (LUMB) muscle strain, which was greater in manual driving; just as muscular strain was greater in assisted driving compared to semi-automatic.

In traditional driving, a tractor driver controls the vehicle direction, using both sight and their hands on the wheel, working on an accelerator pedal. This determines the continuous activation of upper limb muscles. In assisted driving, the operator works as in the manual one, but assisted by the monitor that gives them their whereabouts on the field. Therefore, the driver’s actions in traditional and assisted driving modes are similar; however, in manual driving, the driver needs several reference points to check on the accuracy of the trajectory, entailing the increased movements of their head, trunk, and upper limbs [34]. This accounts for the greater muscular strain, even though not significant, in manual driving than in the assisted one.

Instead, in the semi-automatic driving, the operator pays attention only during the tractor’s turns, without having to keep their hands on the wheel. This assumes lower muscular activation and a consequently reduced muscle strain. Muscle contraction, especially when lasting, may generate a reduced blood flow, getting catabolites to condense, which, together with the biomechanical joints’ overload can cause musculoskeletal disturbances in the long run [23,33,35,36].

The barometric analysis showed that the traditional (manual) driving generated greater Pavg, Pmax, and NC130_400 than the other driving modes. These pressures were reduced with assisted driving but tended to increase with semi-automatic driving.

In semi-automatic driving, the driver tends to not use the wheel, which allows them to make movements other than the ordinary ones, i.e., assuming more resting postures; this leads to a higher pressure of the body on the seat and then on the barometric matrix. Previous studies have shown that a balanced distribution of the operator’s weight on the seat is essential to prevent muscle problems [8]. A prolonged pressure imbalance can result in muscle fatigue and discomfort in the operator during long periods of sitting. Most of the sitting bodyweight must be supported at the ischial tuberosities and must decrease towards the surrounding areas [9].

The highest average speed as of Kinect data was found in the head (joint 4, more than 0.11 m/s), followed by the speed of other joints (joint 3, neck; joint 5, left shoulder; joint 9, right shoulder), from 0.072 to 0.090 m/s.

Except for the right shoulder (joint 9), the maximum speed value was significantly higher during the semi-automatic driving system compared to what was measured during traditional and assisted driving modes. This is probably since the driver did not hold the wheel, liberating the upper body. On the contrary, since the right arm (joint 9) is normally leaning to the armrest of the seat close to the main tractor commands, the right shoulder movements were more constrained, eliciting similar speed values for all driving methods (0.079, 0.078, and 0.078 m/s for manual, assisted and semi-automatic guidance, respectively).

Statistical analysis of the speed values of the joints showed no significant differences between the traditional and assisted driving in all joints, whilst there was a significant difference between manual and assisted driving with the semi-automatic driving for neck and head (joint 3 and 4, respectively). We believe that keeping hands off the wheel dramatically increases the movements of the head and neck caused by the tractor’s vibrations and quakes, with consequent greater stress on the articular structures.

In addition, for manual and assisted driving, the speed of the neck (joints 3) and the head (joints 4) increased over working time, reaching the maximum values at the end of the test. But a significant increase in speed was observed in the final part of the test compared to the beginning and the intermediate part only for the head. This is probably due to a progressively reduced tension (isometric contraction) of head and neck muscles, which, if they are tense at first, soon lose their tone [25,33,37].

It should be specified that, when using the semi-automatic driving system, although the joint speed of the neck and head were almost constant during the entire field test, the observed values of the head joint were significantly greater than the other driving modes. This again supports the evidence that, when the tractor driver does not hold the steering wheel, the upper part of the body is prone to sudden movements at relatively high velocity, likely increasing the physical stress during field operation.

Regarding AcD between the resting and working position, our analysis showed no significant differences between the values obtained for every joint during the field tests carried out using the three driving systems. Nevertheless, both for neck and head, the accumulated distance was higher using semi-automatic driving system than when adopting manual and assisted systems. This data supports previous considerations about the possible improper posture that the tractor driver could exhibit when the semi-automatic driving system is active. In addition, higher advancement speed in semi-automatic driving may be one of the causes of joints and AcD speed increase compared to traditional and assisted driving modes.

It is known that lasting, repetitive movements of joints and continuous vibrations may act together and generate microtraumas to articular structures, thus causing anatomical dysfunctions and even pathologies. At a cervical spine level, this can lead to arthritic events, where the interdiscal fibrous ring loses its resilience, and disc protrusions or hernias may occur [38,39,40].

Of note, both in the USA and Europe, osteoarticular pathologies are the major causes of occupational diseases in farming [41,42,43]. However, despite wide debate around biomechanical overload-related issues of the upper limbs, little is known about the risk factors, incidence, and prevalence of the occupational pathology of the cervical spine.

Lumbo-sacral rachis-related pathologies resulting from the whole body vibration in drivers have been well described [44,45,46]. Further studies will have to be carried out to better understand the effects of vibration transmitted to the entire body, and more generally, of the driving activity on the cervical tract of the spine.

Therefore, considering the evaluation of the upper limbs’ muscular strain, the operator’s pressure on the tractor seat, and the posture assumed by the operator during three different types of driving, we conclude that traditional driving is particularly stressful on the upper limbs muscle engagement. Assisted driving likely generates remarkable muscle activation with pressure forces on the seat and mild joint overload. The semi-automatic mode, as opposed to two alternative methods, requires minimum muscle strain, but greater pressure on the seat. Therefore, assisted driving is the system that causes less discomfort and, in the long run, might result in fewer musculoskeletal and osteoarticular problems in drivers.

The strength of this study was the ability to analyze field observations during harrowing procedures as an alternative to lab tests. The limitation of this analysis was the inability to perform clinical assessments over an extended period, thus associating our field findings with clinical outcomes. In addition, the small number of included workers and the inability to measure vibration transmitted to the entire body should also be considered as limitations of our study.

With regard to the opportunity to associate this study with long-term clinical results, it will be possible, in future trials, to cross-reference the information collected about the stresses during agricultural operations and the available clinical cases. Although it could constitute a limitation of the survey, the limited number of operators was still acceptable thanks to the low value of variability in the data collected between the operators, which made it possible not to consider the difference between operators as significant.

## 5. Conclusions

Assisted driving has a reduced muscular impact, lower pressure on the barometric matrix, and smaller mechanical strain on joints compared to the alternative two driving modes in this study. Furthermore, this driving mode can improve the efficiency of the harrowing operation in terms of process precision. Semi-automatic driving showed better results in the harrowing speed and time needed to complete the assignment, but provoked greater joint stress on the driver’s head and neck.

Kinect turned out to be an excellent tool for the ergonomic analysis in the field. More studies are needed to assess different suspension and seat padding in order to reduce discomfort during semi-automatic driving.

Furthermore, we encourage the use of two armrests to prevent the asymmetrical effect in the joints examined.

## Figures and Tables

**Table 1:** Mean frequency of the signal values (MNF) obtained through the electromyographic system.

Test	AR_LX	SH_LX	SH_RX	LUMB
Manual	7.33 ± 1.65 ^a^	6.55 ± 1.23 ^a^	6.11 ± 1.36 ^a^	3.11 ± 0.78 ^a^
Assisted	5.44 ± 1.74 ^a^	4.22 ±1.09 ^ab^	3.77 ± 1.09 ^ab^	2.22 ± 0.97 ^a^
Semi-automatic	1.33 ± 1.65 ^b^	0.66 ± 1.11 ^b^	0.44 ± 0.72 ^b^	0.11 ± 0.33 ^b^

Note: AR_LX (left arm); SH_LX (left shoulder); SH-RX (right shoulder); LUMB (vertebral column). Letters (a and b) refer to Tukey’s test.

**Table 2:** Pressure values obtained through the barometric system.

Test	Pavg g/cm^2^ (kPa)	Pmax g/cm^2^ (kPa)	NC130_400
Manual	170.11 ± 7.92 (16.7 ± 0.8) ^a^	393.88 ± 54.45 (38.6 ± 5.3) ^a^	0.29 ± 0.04 ^a^
Assisted	151.33 ± 6.30 (14.8 ± 0.6) ^b^	319.00 ± 32.89 (31.2 ± 3.1) ^b^	0.20 ± 0.03 ^b^
Semi-automatic	158.00 ± 6.02 (15.5 ± 0.6) ^b^	360.55 ± 20.64 (35.4 ± 2.0) ^ab^	0.24 ± 0.04 ^ab^

Note: NC130_400 (average percentage of cells activated by pressure values ranging between 130–400 g/cm^2^). Letters (a and b) refer to Tukey’s test.

**Table 3:** The average joint speed (m/s) measured in the four joint points during the field tests.

Guidance	Joint 3	Joint 4	Joint 5	Joint 9
Traditional	0.075 ± 0.045 ^a^	0.124 ± 0.066 ^a^	0.069 ± 0.038 ^a^	0.079 ± 0.047 ^a^
Assisted	0.073 ± 0.035 ^a^	0.116 ± 0.054 ^a^	0.072 ± 0.036 ^ab^	0.078 ± 0.032 ^a^
Semi-automatic	0.090 ± 0.045 ^b^	0.143 ± 0.067 ^b^	0.077 ± 0.039 ^b^	0.078 ± 0.034 ^a^

Letters (a and b) refer to Tukey’s test.

**Table 4:** The average AcD (m) measured in the four joint points between the resting and the working positions during the field tests.

Driving	Joint 3	Joint 4	Joint 5	Joint 9
Traditional	43.73 ± 17.31 ^a^	73.64 ± 28.76 ^a^	40.45 ± 16.06 ^a^	46.00 ± 16.47 ^a^
Assisted	42.83 ± 14.27 ^a^	68.33 ± 25.32 ^a^	42.33 ± 16.92 ^ab^	45.49 ± 11.61 ^a^
Semi-automatic	58.89 ± 18.48 ^b^	85.17 ± 27.43 ^b^	46.46 ± 19.69 ^b^	47.11 ± 14.84 ^a^

Letters (a and b) refer to Tukey’s test.

## References

[1] Ugochukwu A.I., Phillips P.W. (2018). Technology Adoption by Agricultural Producers: A Review of the Literature. From Agriscience to Agribusiness.

[2] Triantafyllou A., Sarigiannidis P., Bibi S. (2019). Precision Agriculture: A Remote Sensing Monitoring System Architecture & Dagger. Info.

[3] Antille D.L., Peets S., Galambošová J., Botta G.F., Rataj V., Macak M., Tullberg J.N., Chamen W.C.T., White D.R., Misiewicz P.A. (2019). Soil compaction and controlled traffic farming in arable and grass cropping systems. Agron. Res..

[4] Hargreaves P.R., Peets S., Chamen W.C.T., White D.R., Misiewicz P.A., Godwin R.J. (2019). Improving grass silage production with controlled traffic farming (CTF): Agronomics, system design and economics. Precis. Agric..

[5] Bergtold J.S., Raper R., Schwab E.B. (2009). The Economic Benefit of Improving the Proximity of Tillage and Planting Operations in Cotton Production with Automatic Steering. Appl. Eng. Agric..

[6] Ortiz B., Balkcom K.B., Duzy L., Van Santen E., Hartzog D.L. (2013). Evaluation of agronomic and economic benefits of using RTK-GPS-based auto-steer guidance systems for peanut digging operations. Precis. Agric..

[7] Dey A.K., Mann D.D. (2020). Workload Associated with Operation of an Agricultural Sprayer. Contemp. Ergon..

[8] Romano E., Pirozzi M., Ferri M., Calcante A., Oberti R., Vitale E., Rapisarda V. (2020). The use of pressure mapping to assess the comfort of agricultural machinery seats. Int. J. Ind. Ergon..

[9] Metha C., Pandey M., Tiwari P., Gite L., Majumder J. (2007). Design of Tractor Controls Based on Ergonomical Considerations. Developments in Agricultural and Industrial Ergonomics: General Studies.

[10] Krüger J. (2019). Influence of posture on the deviation of measured acceleration values for tractor operators. Biosyst. Eng..

[11] Brookhuis K.A., De Waard D. (2010). Monitoring drivers’ mental workload in driving simulators using physiological measures. Accid. Anal. Prev..

[12] Tuure V.-M. (1992). Determination of physical stress in agricultural work. Int. J. Ind. Ergon..

[13] Fairley T.E. (1995). Predicting the discomfort caused by tractor vibration. Ergonomics.

[14] Futatsuka M., Maeda S., Inaoka T., Nagano M., Shono M., Miyakita T. (1998). Whole-Body Vibration and Health Effects in the Agricultural Machinery Drivers. Ind. Health.

[15] Morgan L., Mansfield N.J. (2014). A survey of expert opinion on the effects of occupational exposures to trunk rotation and whole-body vibration. Ergonomics.

[16] Mayton A., Kittusamy N., Ambrose D., Jobes C., Legault M. (2008). Jarring/jolting exposure and musculoskeletal symptoms among farm equipment operators. Int. J. Ind. Ergon..

[17] Jing Y., Liu G., Xia Y. (2019). Automatic navigation system and control methods based on GNSS-controlled land leveling technology. Int. Agr. Eng. J..

[18] Phinyomark A., Limsakul C., Phukpattaranont P. (2009). A Novel Feature Extraction for Robust EMG Pattern Recognition. J. Comput..

[19] Cifrek M., Medved V., Tonković S., Ostojić S. (2009). Surface EMG based Muscle Fatigue Evaluation in Biomechanics. Clin. Biomech..

[20] (2012). Computational Intelligence in Electromyography Analysis—A Perspective on Current Applications and Future Challenges. Computational Intelligence in Electromyography Analysis–A Perspective on Current Applications and Future Challenges.

[21] Du S., Vuskovic M. Temporal vs. spectral approach to feature extraction from prehensile EMG signals. Proceedings of the 2004 IEEE International Conference on Information Reuse and Integration.

[22] Farina D., Merletti R. (2003). A novel approach for estimating muscle fiber conduction velocity by spatial and temporal filtering of surface emg signals. IEEE Trans. Biomed. Eng..

[23] González-Izal M., Rodríguez-Carreño I., Malanda A., Mallor-Giménez F., Navarro-Amézqueta I., Gorostiaga E., Izquierdo M. (2010). sEMG wavelet-based indices predicts muscle power loss during dynamic contractions. J. Electromyogr. Kinesiol..

[24] Piper H. (1912). Elektrophysiologie Menschlicher Muskeln.

[25] Valentino M., Rapisarda V., Scalise L., Paone N., Santarelli L., Fenga C., Rossi G.L. (2004). A new method for the experimental assessment of finger haemodynamic effects induced by a hydraulic breaker in operative conditions. J. Occup. Health.

[26] Pirozzi M., Rapisarda V., Ferri M., Calcante A., Oberti R., Romano E. (2017). A study of a barometric methodology for assessing the agricultural and forestry machine’s seat comfort. Chem. Eng. Trans..

[27] Otte K., Kayser B., Mansow-Model S., Verrel J., Paul F., Brandt A.U., Schmitz-Hübsch T. (2016). Accuracy and Reliability of the Kinect Version 2 for Clinical Measurement of Motor Function. PLoS ONE.

[28] Lachat E., Macher H., Mittet M.-A., Landes T., Grussenmeyer P. (2015). First Experiences with Kinect V2 Sensor for Close Range 3D Modelling. ISPRS.

[29] Arai K., Andrie R. (2013). 3D Skeleton model derived from Kinect Depth Sensor Camera and its application to walking style quality evaluations. Int. J. Adv. Res. Artif. Intell..

[30] Li R., Si W., Weinmann M., Klein R. (2019). Constraint-Based Optimized Human Skeleton Extraction from Single-Depth Camera. Sensors.

[31] Marinello F., Pezzuolo A., Simonetti A., Grigolato S., Boscaro D., Mologni O., Gasparini F., Cavalli R., Sartori L. (2015). Tractor cabin ergonomics analyses by means of kinect motion capture technology. Contemp. Eng. Sci..

[32] Karimi D., Henry J., Mann D.D. (2012). Effect of Using GPS Autosteer Guidance Systems on the Eye-Glance Behaviour and Posture of Tractor Operators. J. Agr. Saf. Health.

[33] Romano E., Caruso L., Longo D., Vitale E., Schillaci G., Rapisarda V. (2019). Investigation of hand forces applied to a pruning tool–pilot study. Ann. Agric. Environ. Med..

[34] Mozafari A., Vahedian M., Mohebi S., Najafi M. (2015). Work-related musculoskeletal disorders in truck drivers and official workers. Acta Med. Iran..

[35] Allen D.G., Lamb G.D., Westerblad H. (2008). Skeletal Muscle Fatigue: Cellular Mechanisms. Physiol. Rev..

[36] Cashaback J.G., Cluff T., Potvin J.R. (2013). Muscle fatigue and contraction intensity modulates the complexity of surface electromyography. J. Electromyogr. Kinesiol..

[37] Vitale E., Ledda C., Adani R., Lando M., Bracci M., Cannizzaro E., Tarallo L., Rapisarda V. (2019). Management of High-Pressure Injection Hand Injuries: A Multicentric, Retrospective, Observational Study. J. Clin. Med..

[38] Lan F.-Y., Liou Y.-W., Huang K.-Y., Guo H., Wang J.-D. (2016). An investigation of a cluster of cervical herniated discs among container truck drivers with occupational exposure to whole-body vibration. J. Occup. Health.

[39] Stamatiadis N., Pappalardo G., Cafiso S. (2017). Use of technology to improve bicycle mobility in smart cities. 5th IEEE International Conference on Models and Technologies for Intelligent Transportation Systems.

[40] Charles L.E., Ma C.C., Burchfiel C.M., Dong R.G. (2018). Vibration and Ergonomic Exposures Associated With Musculoskeletal Disorders of the Shoulder and Neck. Saf. Health Work..

[41] Davis K.G., Kotowski S.E. (2007). Understanding the ergonomic risk for musculoskeletal disorders in the United States agricultural sector. Am. J. Ind. Med..

[42] Gómez-Galán M., Pérez-Alonso J., Callejón-Ferre Á.J., López-Martínez J. (2017). Musculoskeletal disorders: OWAS review. Ind. Health.

[43] Bosch L.M., Van Der Molen H.F., Frings-Dresen M.H. (2018). Optimizing implementation of interventions in agriculture for occupational upper extremity musculoskeletal disorders: Results of an expert panel. Work.

[44] Wolfgang R., Burgess-Limerick R. (2014). Whole-body vibration exposure of haul truck drivers at a surface coal mine. Appl. Ergon..

[45] Wahlström J., Burström L., Johnson P.W., Nilsson T., Järvholm B. (2018). Exposure to whole-body vibration and hospitalization due to lumbar disc herniation. Int. Arch. Occup. Environ. Health.

[46] Lukman K.A., Jeffree M.S., Rampal K.G. (2018). Lower back pain and its association with whole-body vibration and manual materials handling among commercial drivers in Sabah. Int. J. Occup. Saf. Ergon..

